# Association of visit-to-visit HbA1c variability with cardiovascular diseases in type 2 diabetes within or outside the target range of HbA1c

**DOI:** 10.3389/fpubh.2022.1052485

**Published:** 2022-11-10

**Authors:** Bao Sun, Yongchao Gao, Fazhong He, Zhaoqian Liu, Jiecan Zhou, Xingyu Wang, Wei Zhang

**Affiliations:** ^1^Department of Clinical Pharmacology, Xiangya Hospital, Central South University, Changsha, China; ^2^Department of Pharmacy, The Second Xiangya Hospital, Central South University, Changsha, China; ^3^Institute of Clinical Pharmacy, Central South University, Changsha, China; ^4^Institute of Clinical Pharmacology, Central South University, Hunan Key Laboratory of Pharmacogenetics, Changsha, China; ^5^Engineering Research Center of Applied Technology of Pharmacogenomics, Ministry of Education, Changsha, China; ^6^National Clinical Research Center for Geriatric Disorders, Changsha, China; ^7^Department of Pharmacy, Zhuhai People's Hospital (Zhuhai Hospital Affiliated With Jinan University), Zhuhai, China; ^8^The First Affiliated Hospital, Clinical Medical Research Center, Hengyang Medical School, University of South China, Hengyang, China; ^9^The First Affiliated Hospital, Hengyang Clinical Pharmacology Research Center, Hengyang Medical School, University of South China, Hengyang, China; ^10^Beijing Hypertension League Institute, Beijing, China

**Keywords:** HbA1c variability, type 2 diabetes, major microvascular events, within the target range of HbA1c, outside the target range of HbA1c

## Abstract

**Background:**

Although a growing attention has been recently paid to the role of HbA1c variability in the risk of diabetic complications, the impact of HbA1c variability on cardiovascular diseases (CVD) in type 2 diabetes is still debated. The aim of the study is to investigate the association of HbA1c variability with CVD in individuals within or outside the target range of HbA1c.

**Methods:**

Using data from Action in Diabetes and Vascular disease: preterAx and diamicroN-MR Controlled Evaluation (ADVANCE), we enrolled 855 patients with type 2 diabetes in China. The primary outcomes included major macrovascular events and major microvascular events. Visit-to-visit HbA1c variability was expressed as the coefficient of variation (CV) of five measurements of HbA1c taken 3–24 months after treatment. Cox proportional hazard models were used to estimate adjusted hazard ratios (aHR).

**Results:**

Among 855 patients in the intensive glucose treatment group, 563 and 292 patients were assigned to the group of “within the target range of HbA1c” (WTH) (updated mean HbA1c ≤ 7.0%) and “outside the target range of HbA1c” (OTH) (updated mean HbA1c > 7.0%), respectively. HbA1c variability was positively associated with the risk of major microvascular events in all patients and both the subgroups during a median follow-up period of 4.8 years. Particularly, the risk related to HbA1c variability was higher in patients in WTH group for the new or worsening nephropathy [aHR: 3.35; 95% confidence interval (CI): 1.05–10.74; *P* = 0.042].

**Conclusions:**

This retrospective cohort study confirmed the positive correlation between HbA1c variability and major microvascular events, especially in subjects in WTH or OTH.

## Introduction

Diabetes is a major driver of cardiovascular diseases (CVD) and mortality worldwide (1), and associations between glycemic control and CVD are still debated (2). Although HbA1c, an integral marker of glycemic exposure over the past 2–3 months, has become the gold standard to assess glycemic control, it cannot describe interday or intraday glucose fluctuations. In recent years, a growing attention has been paid to the important role of glycemic variability (GV) in the development of CVD (3–5). GV refers to oscillations in blood glucose levels over a certain interval of time. The widespread use of continuous glucose monitoring (CGM) and self-monitored blood glucose (SMBG) technology has provided the opportunity to assess short-term GV (both within-day and between-day GV) and long-term GV (based on serial determinations in blood glucose over a longer period of time) (6). A meta-analysis indicated that HbA1c variability was superior at predicting diabetic complications than mean HbA1c (7). Characterized by its simplicity for glucose concentration, coefficient of variation (%CV) is considered as the most appropriate indicator for assessing GV because it is easy to calculate and is independent of the mean glucose level (8).

Many observational studies and randomized controlled trials suggested that GV was closely associated with CVD in patients with type 1 or type 2 diabetes mellitus (T2DM) (7, 9–17). However, there were several heterogeneous results regarding the association between GV and diabetic complications (6, 18–20). A recent study revealed that HbA1c variability seemed to play a greater role in microvascular complications among patients with relatively optimal baseline glycemic control (21). Nevertheless, there was not yet complete understanding of the possible impact of HbA1c variability on CVD in patients within or outside the recommended HbA1c target (22). Given that the current guidelines from the Chinese Diabetes Society and American Diabetes Association recommend an HbA1c level of <7.0% as the treatment goal (23, 24), we explore the impact of HbA1c variability on the development of CVD among patients within or outside the recommended HbA1c target.

## Methods

### Study design and participants

This study was part of the ADVANCE, a factorial randomized and controlled trial of intensive blood glucose and blood pressure lowering treatment in patients with type 2 diabetes (25). The patients were enrolled from 61 centers in China, and the details of these patients had been described in our previous study (26). The study was approved by each center's institutional review board, and all participants provided written informed consent.

Patients were randomly assigned (1:1) to receive modified release gliclazide-based intensive or standard therapy for glycemic control, and perindopril-indapamide or matching placebo for blood pressure control. The intensive glucose control received gliclazide-modified release-based strategy (target HbA1c ≤ 6.5%).

### Measurement of visit-to-visit HbA1c variability

Fasting HbA1c samples were collected at baseline, at 3, 6, 12, 18, 24 months, and every 6 months thereafter in the intensive glucose treatment group. Standard glucose treatment group was not included in our study, due to insufficient measurements taken (fasting HbA1c was only measured at 6, 12, and 24 months during the follow up of first 2 years). To eliminate the effect of multiple HbA1c measures in a short space of time, we used the mean value of serially measured HbA1c in each participant. The %CV of HbA1c was calculated as the standard deviations (SD) divided by the updated mean value of HbA1c [%CV = (SD/mean HbA1c) × 100], which was independent of the mean glucose level. Based on the previous studies (15, 27), participants were divided into high HbA1c variability and low HbA1c variability for further analyses.

### Study outcomes and follow-up

The primary outcomes included major macrovascular events and major microvascular events. The major microvascular events comprised of new or worsening nephropathy or retinopathy, which was considered as the secondary outcomes. A previous study clearly described the primary and secondary outcomes (28). The duration of follow-up for each participant ranged from their 24-month visit until they experienced events, deaths, or completed the final visit at the end of the study.

### Statistical analysis

Data are summarized as means ± SD for continuous variables and as percentages for categorical variables. Baseline clinical characteristics were compared with the use of Student's *t*-test, Wilcoxon rank sum test or χ^2^ tests. Multivariable Cox regression analyses was used to explore the association between HbA1c variability and the risk of CVD. A backward stepwise was utilized for baseline covariates in the multivariable model including age, duration of diabetes, gender, body mass index (BMI), current smoking status, systolic and diastolic blood pressure, total cholesterol, triglycerides, high- and low-density lipoprotein cholesterol, history of major macrovascular diseases and microvascular diseases, baseline use of insulin and mean HbA1c during the first 24 months. In addition, stratified analyses were performed for patients with average HbA1c levels ≤ 7.0% or > 7.0% during the follow-up. High HbA1c variability and low HbA1c variability was estimated separately for these two subgroups. Kaplan–Meier estimates were employed to compare the freedom from CVD within groups defined by HbA1c variability. The SPSS version 20.0 (SPSS Inc., Chicago, IL, USA) was performed for all statistical analyses, and a two-sided *P* value < 0.05 was considered statistically significant.

## Results

**Figure 1:**
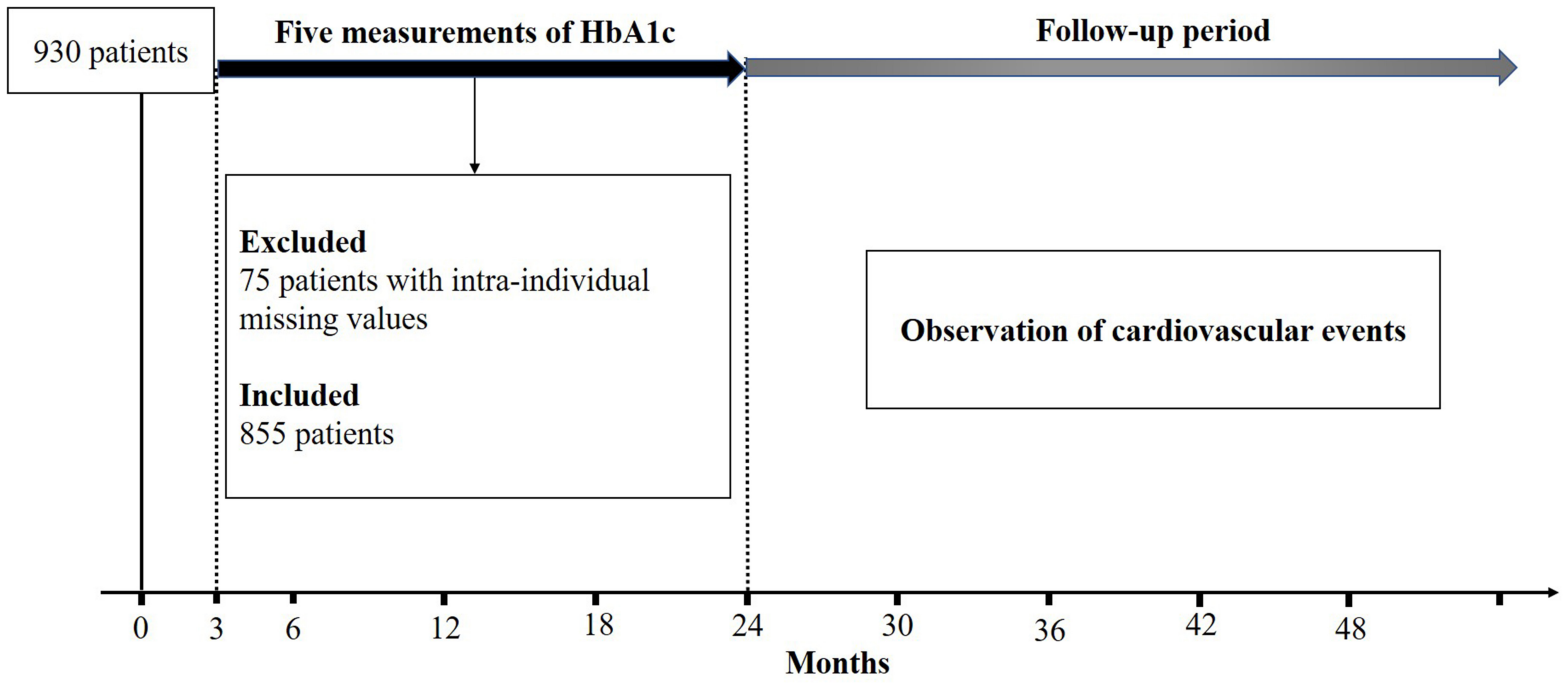
Experimental design in the intensive glucose control.

A total of 855 patients with type 2 diabetes in the intensive glycemic control were included for the final analysis (excluding intra-individual missing values of HbA1c) (Figure 1), including 424 males and 431 females. The detailed clinical characteristics of patients at baseline are described in Table 1. Based on the updated mean of intra-individual HbA1c, 563 (65.8%) and 292 (34.2%) patients were assigned to the group of “within the target range of HbA1c” (WTH) and “outside the target range of HbA1c” (OTH), respectively.

**Table 1:** Characteristics of the study population by the target range of HbA1c in the intensive glucose control.

**Characteristic**	**Intensive glucose control group**		***P*-value**
	**WTH (n = 563)**	**OTH (n = 292)**	
Age (years)	65.08 ± 5.91	64.15 ± 5.87	0.03
Gender (males, %)	50.30	48.30	0.58
Smoking, %	22.40	26.40	0.19
BMI (kg/m^2^)	25.51 ± 3.26	25.11 ± 3.17	0.09
Duration of diabetes (years)	7.04 ± 6.01	9.27 ± 5.92	<0.001
History of major macrovascular disease, %	18.12	18.84	0.79
History of major microvascular disease, %	12.80	13.01	0.93
HbA1c, %	6.30 ± 0.43	7.90 ± 0.81	<0.001
SD of HbA1c	0.38 ± 0.27	0.82 ± 0.48	<0.001
CV of HbA1c, %	6.08 ± 4.50	10.10 ± 5.55	<0.001
Systolic blood pressure (mmHg)	138.48 ± 21.42	139.57 ± 21.18	0.48
Diastolic blood pressure (mmHg)	78.04 ± 10.65	79.53 ± 11.97	0.06
Total cholesterol (mmol/l)	5.33 ± 1.39	5.47 ± 1.21	0.17
Triglycerides (mmol/l)	1.96 ± 2.05	2.14 ± 1.64	0.19
HDL cholesterol (mmol/l)	1.29 ± 0.48	1.36 ± 0.55	0.05
LDL cholesterol (mmol/l)	3.37 ± 4.19	3.64 ± 5.71	0.43
Insulin, %	49.00	82.90	<0.001
Metformin, %	58.30	68.50	0.004
Calcium channel blockers, %	36.80	29.10	0.025
α-glucosidase inhibitors, %	59.90	68.80	0.01

WTH, within the target range of HbA1c; OTH, outside the target range of HbA1c; BMI, body mass index; SD, standard deviations; CV, coefficient of variation; HDL, high-density lipoprotein; LDL, low-density lipoprotein.

The %CV of HbA1c was significantly lower in group of WTH (6.08 ± 4.50) than those in OTH (10.10 ± 5.55) during a median follow-up period of 4.8 years. The median value of %CV of HbA1c was used to define high and low HbA1c variability.

### Effects of visit-to-visit HbA1c variability on the development of CVD

**Figure 2:**
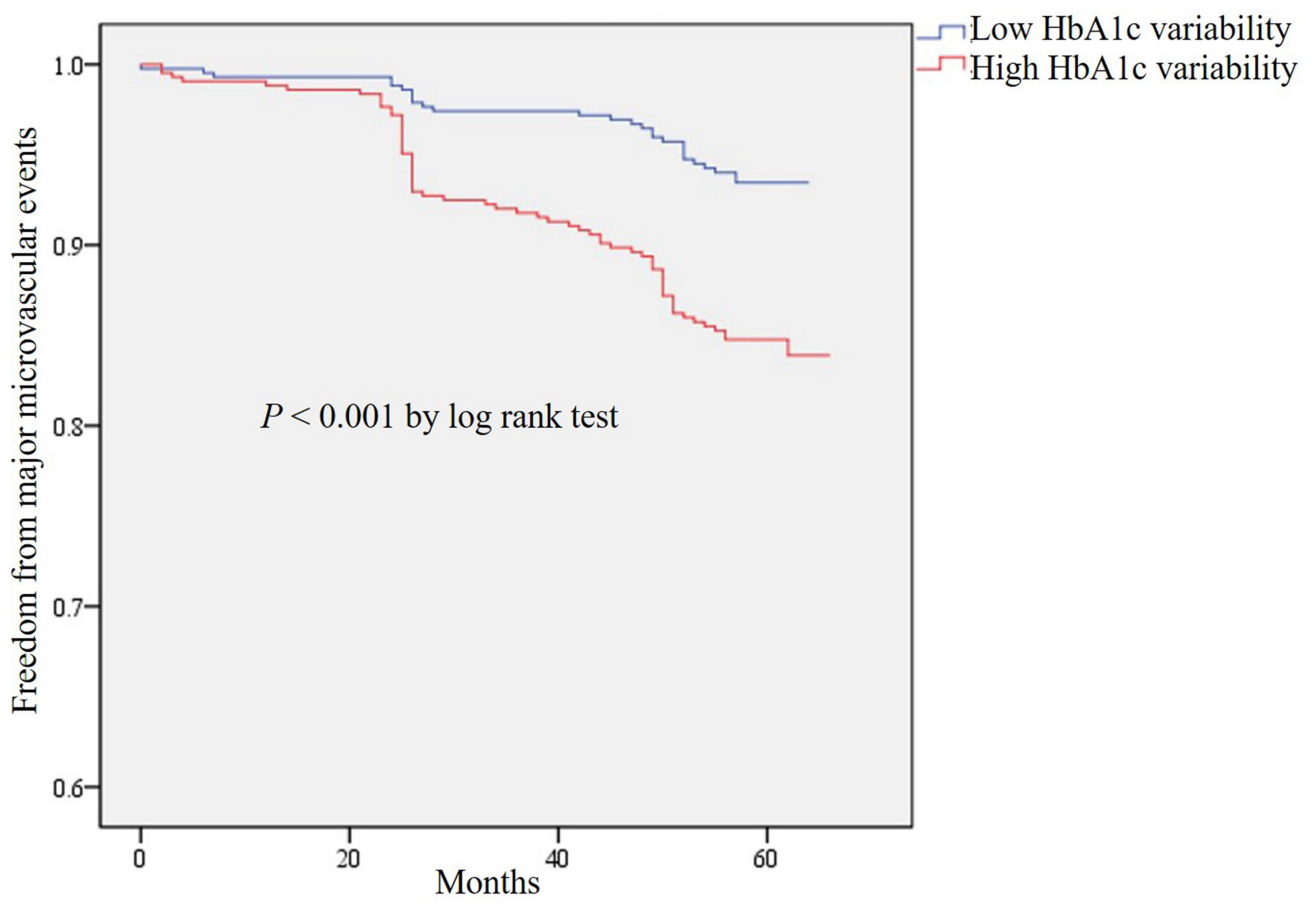
Kaplan–Meier curves of freedom from major microvascular events for HbA1c variability in total patients.

Compared with patients with the low HbA1c variability, the risk of major microvascular events was significantly increased in patients with the high HbA1c variability (aHR = 1.97, 95% CI 1.18–3.30, *P* = 0.010) after adjusting the potential confounding factors (Table 2). However, there was no association between high HbA1c variability and major macrovascular events. The Kaplan–Meier plot of freedom from major microvascular events between low and high HbA1c variability was presented in Figure 2. In addition, we further performed the multiple regression analyses for secondary outcomes and found that aHRs for new or worsening nephropathy and retinopathy were 3.12 (95% CI 1.32–7.35, *P* = 0.0090) and 1.75 (95% CI 0.96–3.17, *P* = 0.066), respectively, in patients with high HbA1c variability (Table 2).

**Table 2:** The risk of cardiovascular events according to visit-to-visit HbA1c variability in all patients.

**Cardiovascular events**	**Low HbA1c variability**	**High HbA1c variability**	***P*-value**
Major microvascular events			
No. of cases	27	67	
aHR*^a^* (95% CI)	1 (Reference)	1.97 (1.18–3.30)	**0.010**
New or worsening nephropathy			
No. of cases	8	27	
aHR*^*a*^* (95% CI)	1 (Reference)	3.12 (1.32–7.35)	**0.0090**
New or worsening retinopathy			
No. of cases	20	51	
aHR*^*a*^* (95% CI)	1 (Reference)	1.75 (0.96–3.17)	0.066
Major macrovascular events			
No. of cases	51	53	
aHR*^*a*^* (95% CI)	1 (Reference)	1.22 (0.77–1.92)	0.39
All-cause death			
No. of cases	17	19	
aHR*^*a*^* (95% CI)	1 (Reference)	0.92 (0.43–1.97)	0.83

aHR, adjusted hazard ratios; CI, confidence interval.

^a^adjusted for age, duration of diabetes, gender, BMI, current smoking status, systolic and diastolic blood pressure, total cholesterol, triglycerides, high- and low-density lipoprotein cholesterol, baseline use of insulin and mean HbA1c during the first 24 months, history of major macrovascular diseases and microvascular diseases. The meaning of the bold values represent that the values reach significant level.

### Subgroup analyses in patients within or outside the target range of HbA1c

Subgroup analyses were performed using multivariable Cox regression analyses in subjects of WTH and OTH group (Tables 3, 4). In general, the associations of HbA1c variability with the risk of major microvascular events were of statistical significance in both patients of WTH and OTH group. In WTH group, high HbA1c variability increased the risk of developing major microvascular events by 2.20 folds (Table 3), and high HbA1c variability increased such risk by 2.26 folds in OTH group (Table 4). The Kaplan–Meier plot of freedom from major microvascular events between low and high HbA1c variability in subgroup analyses was presented in Supplementary Figure 1. Of note, this consistent trend was also found for new or worsening nephropathy in patients of WTH group.

**Table 3:** The risk of cardiovascular events according to visit-to-visit HbA1c variability in patients of WTH group.

**Cardiovascular events**	**Low HbA1c variability**	**High HbA1c variability**	***P*-value**
Major microvascular events			
No. of cases	13	31	
aHR*^*a*^* (95% CI)	1 (Reference)	2.20 (1.13–4.28)	**0.020**
New or worsening nephropathy			
No. of cases	3	14	
aHR*^*a*^* (95% CI)	1 (Reference)	3.35 (1.05–10.74)	**0.042**
New or worsening retinopathy			
No. of cases	10	21	
aHR*^*a*^* (95% CI)	1 (Reference)	1.80 (0.84–3.88)	0.13
Major macrovascular events			
No. of cases	35	36	
aHR*^*a*^* (95% CI)	1 (Reference)	1.04 (0.61–1.75)	0.39
All-cause death			
No. of cases	14	8	
aHR*^*a*^* (95% CI)	1 (Reference)	0.58 (0.23–1.45)	0.24

WTH, within the target range of HbA1c; aHR, adjusted hazard ratios; CI, confidence interval.

^a^adjusted for age, duration of diabetes, gender, BMI, current smoking status, systolic and diastolic blood pressure, total cholesterol, triglycerides, high- and low-density lipoprotein cholesterol, baseline use of insulin and mean HbA1c during the first 24 months, history of major macrovascular diseases and microvascular diseases. The meaning of the bold values represent that the values reach significant level.

**Table 4:** The risk of cardiovascular events according to visit-to-visit HbA1c variability in patients of OTH group.

**Cardiovascular events**	**Low HbA1c variability**	**High HbA1c variability**	***P* value**
Major microvascular events			
No. of cases	15	35	
aHR*^*a*^* (95% CI)	1 (Reference)	2.26 (1.09–4.69)	**0.029**
New or worsening nephropathy			
No. of cases	5	13	
aHR*^*a*^* (95% CI)	1 (Reference)	2.53 (0.81–7.90)	0.11
New or worsening retinopathy			
No. of cases	14	26	
aHR*^*a*^* (95% CI)	1 (Reference)	1.53 (0.68–3.41)	0.30
Major macrovascular events			
No. of cases	16	17	
aHR*^*a*^* (95% CI)	1 (Reference)	1.10 (0.50–2.42)	0.81
All-cause death			
No. of cases	8	6	
aHR*^*a*^* (95% CI)	1 (Reference)	0.56 (0.15–2.06)	0.38

OTH, outside the target range of HbA1c; aHR, adjusted hazard ratios; CI, confidence interval.

^a^adjusted for age, duration of diabetes, gender, BMI, current smoking status, systolic and diastolic blood pressure, total cholesterol, triglycerides, high- and low-density lipoprotein cholesterol, baseline use of insulin and mean HbA1c during the first 24 months, history of major macrovascular diseases and microvascular diseases. The meaning of the bold values represent that the values reach significant level.

## Discussion

In the present study, we found that high HbA1c variability was associated with risk of major microvascular events in patients with T2DM after long-term follow-up. Particularly, our result highlighted the importance of HbA1c variability even in patients within or outside the target range of HbA1c.

The possible role of HbA1c variability in the development of CVD is still a remaining unanswered question in T2DM. Several studies suggested an association between HbA1c variability and CVD (14, 16, 17). In contrast, others showed no association of HbA1c variability with cardiovascular outcomes (18–20). Interestingly, a previous study also indicated that HbA1c variability (SD of HbA1c) was correlated with combined macro/microvascular events and macrovascular events, but not with microvascular events (10). In this study, we revealed an association of HbA1c variability (%CV of HbA1c) with major microvascular events in patients regardless of being within the target range of HbA1c, but not with major macrovascular events. One of the possible explanations for such disparity may be due to the absence of standardized definitions for HbA1c variability. For instance, EI Malahi et al. (29) demonstrated that GV [assessed by time in range (TIR)] was independently associated with the presence of composite microvascular complications, while it (assessed by TIR, SD, and CV) did not show a link with macrovascular complications. Thus, further research defining the standardized GV is required to elucidate these controversial results.

On the other hand, several related confounding factors might be involved in CVD. Drug therapy including insulin, metformin, α-glucosidase inhibitors had direct or indirect impacts on CVD. Pieber et al. (30) found that severe hypoglycemia induced by insulin was significantly associated with the risk of CVD. A recent study provided that metformin could ameliorate the prognosis of heart failure by the modulation of glucose and lipid metabolism, the attenuation of oxidative stress and inflammation, and the inhibition of myocardial cell apoptosis (31). Another study considered that α-glucosidase inhibitors contributed to the significant beneficial CVD outcome *via* affecting endothelial dysfunction and carotid intima media thickening (32). In the present study, there were significant differences in the use of anti-diabetic drugs, which might affect the HbA1c variability and confound the ultimate results. Now-a-days, cholesterol-lowering drugs should also be taken into account in diabetic patients with lipid abnormalities. Gentile et al. found that statins not only reduced low density lipoprotein (LDL)-cholesterol levels, but also resulted in the reduction of inflammation and CVD mortality (33). In this study, we did not provide the information about the statins use, but the LDL-cholesterol levels were not significant differences between WTH and OTH group. In addition, a previous study observed that people with young-onset T2DM had a higher prevalence of diabetic complications than those with late-onset T2DM because of the longer duration of diabetes (34). Similarly, duration of diabetes in OTH group was longer and more significant than that in WTH group, which might also affect the risk of major macrovascular events in our study. Nevertheless, the results remained present after adjusting for these confounding factors in the present study. Alternatively, another likely explanation is that the effect of HbA1c variability may be diluted in the general diabetes patients due to the different related confounding factors.

Several pathways and mechanisms linking HbA1c variability to CVD have been proposed. Endothelial dysfunction, inflammatory cytokines and oxidative stress were proposed as mediators of the HbA1c variability involved in CVD (35, 36). A recent study provided the evidence that oxidative damage was even more serious in glucose variability model than that in prolonged hyperglycemia model (37). Therefore, basic research regarding the elaborated mechanisms of HbA1c variability in the development of CVD is still needed.

The strengths of our study include the use of a database with a long-term follow-up and a large number of HbA1c measurements. Moreover, in addition to the major microvascular events, we further analyze the association of HbA1c variability with new or worsening nephropathy and retinopathy. Inevitably, several limitations to our study should be noted. Since this is a retrospective cohort study, there may be uncorrected confounding factors such as drug therapy and duration of diabetes. To ensure the robustness of the findings, we adjusted the confounding factors in the analyses and performed subgroup analyses. Despite the adjustments for a broad set of confounding factors, we could not exclude the possibility of residual or unmeasured confounding factors. Selection bias may be another limitation due to the exclusion of patients with intra-individual missing values of HbA1c. In addition, the included subjects are from centers in China, which may not be generalizable to other ethnic lines. Also, the sample size is small in our study, especially in the subgroup of OTH, which needs to be verified by large samples in future studies. Finally, we do not investigate the elaborated mechanisms linking HbA1c variability and the risk of major microvascular events, which deserves future experiments to figure them out.

## Conclusions

In conclusion, our study showed that HbA1c variability was an independent risk factor for major microvascular events in T2DM patients within or outside the target range of HbA1c.

## Data availability statement

The original contributions presented in the study are included in the article/Supplementary material, further inquiries can be directed to the corresponding authors.

## Ethics statement

Ethical review and approval was not required for the study on human participants in accordance with the local legislation and institutional requirements. Written informed consent from the patients/participants or patients/participants' legal guardian/next of kin was not required to participate in this study in accordance with the national legislation and the institutional requirements.

## Author contributions

BS contributed to the study design and data analysis and edited the manuscript. YG and FH collected the data and performed the statistical analysis. ZL contributed to revising the manuscript. JZ, XW, and WZ are the guarantor of this work and takes responsibility for the accuracy of the data analysis. All authors reviewed and approved the final submitted report.

## Funding

This study was supported by National Scientific Foundation of China (Nos. 82104307, 81573511, 81874329, 82003872, and 81522048), National Key Research and Development Program (Nos. 2016YFC0905000 and 2016YFC0905001), Natural Science Foundation of Hunan Province (Nos. 2021JJ40865 and 2020JJ5513), Scientific Research Fund Project of Hunan Provincial Health Commission (Nos. 20201973 and B202313016776), Scientific Research Fund of Hunan Provincial Education Department (No. 19A418), Central Government Funds for Guiding Local Scientific and Technological Development (2021QZY016), Hunan Province Clinical Medical Technology Innovation Guidance Project (2020SK51823), and Scientific Research Launch Project of the Second Xiangya Hospital of Central South University.

## Conflict of interest

The authors declare that the research was conducted in the absence of any commercial or financial relationships that could be construed as a potential conflict of interest.

## Publisher's note

All claims expressed in this article are solely those of the authors and do not necessarily represent those of their affiliated organizations, or those of the publisher, the editors and the reviewers. Any product that may be evaluated in this article, or claim that may be made by its manufacturer, is not guaranteed or endorsed by the publisher.

## Supplementary material

Supplementary Figure 1 Kaplan–Meier curves of freedom from major microvascular events for HbA1c variability in subgroup analyses. **(A)** Kaplan–Meier curves of freedom from major microvascular events for HbA1c variability in WTH group. **(B)** Kaplan–Meier curves of freedom from major microvascular events for HbA1c variability in OTH group.

## Abbreviations

CVD: cardiovascular diseases
ADVANCE: Action in Diabetes and Vascular disease: preterAx and diamicroN-MR Controlled Evaluation
CV: coefficient of variation
aHR: adjusted hazard ratios
WTH: within the target range of HbA1c
OTH: outside the target range of HbA1c
CI: confidence interval
GV: glycemic variability
CGM: continuous glucose monitoring
SMBG: self-monitored blood glucose
T2DM: type 2 diabetes mellitus
SD: standard deviations
BMI: body mass index
TIR: time in range
LDL: low density lipoprotein.
