# Supplementary figures and images for: Association of visit-to-visit HbA1c variability with cardiovascular diseases in type 2 diabetes within or outside the target range of HbA1c
Source: Front Public Health. 2022 Nov 10;10:1052485. doi: 10.3389/fpubh.2022.1052485 (PMC9686379; doi:10.3389/fpubh.2022.1052485)

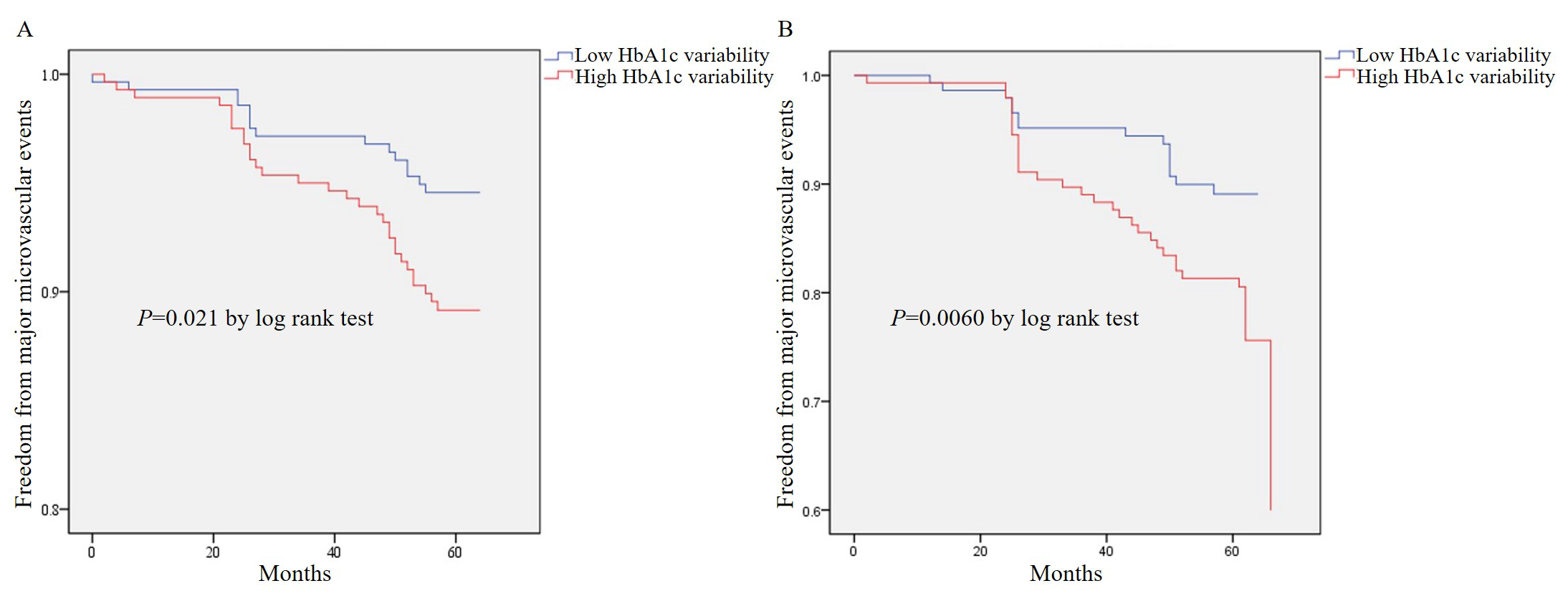

Supplement: Supplementary Figure 1 — Kaplan–Meier curves of freedom from major microvascular events for HbA1c variability in subgroup analyses. (A) Kaplan–Meier curves of freedom from major microvascular events for HbA1c variability in WTH group. (B) Kaplan–Meier curves of freedom from major microvascular events for HbA1c variability in OTH group. [file Image_1.TIF]
